# Editorial Comment: Testicular descent revisited: a micro-computed tomography study in fetal rats

**DOI:** 10.1590/S1677-5538.IBJU.2023.04.05

**Published:** 2023-05-15

**Authors:** Luciano A. Favorito

**Affiliations:** 1 Universidade do Estado do Rio de Janeiro Unidade de Pesquisa Urogenital Rio de Janeiro RJ Brasil Unidade de Pesquisa Urogenital - Universidade do Estado do Rio de Janeiro - Uerj, Rio de Janeiro, RJ, Brasil

Moritz Markel ^1^, Nicole Peukert ^2^, Marco Ginzel ^3^, Rainer Haak ^4^, Steffi Mayer ^2^, Martin Lacher ^2^, et al.

Surg Int. 2023 Mar 8;39(1):149.

## COMMENT

Testicular Migration is a complex process with a great importance for understood the testicular anomalies. This process has two phases: (a) Abdominal stage – Testicular migration from the abdomen to the internal inguinal ring that begins around the 8th WPC and lasts until the 15th WPC and (b) Inguino-scrotal stage - Transition of the testes through the inguinal canal until their definitive arrival in the scrotum that begins around the 20th WPC and lasts until the 30th WPC (1, 2). There are several factor involved in this process and the most important are the hormonal stimulus, the role of intra-abdominal pressure and the development of the gubernaculum testis (3–5). The gubernaculum seems to be the most important anatomical structure in the testicular migration process, by means of contraction and shortening, thus imposing traction strength on the testis and facilitates the transition of the testis by through the inguinal canal (5).

In the present study (6) the authors in an elegant study analyzed the role of the gubernaculum and the development of the processus vaginalis peritonei with Micro-computed tomography (μCT) in rodents. The μCT imaging confirmed the intraperitoneal location of the testicles from from embryonic day 15 to newborns. The components of the inner genital moved closer together while the intestinal volume expanded. The gubernacular bulb seemed to be involved in the formation of the processus vaginalis peritonei. The authors shows in this interesting paper new morphologic aspects on the development of the processus vaginalis peritonei.
